# The effect of child development on the components of the Frequency Following Response: Child development and the Frequency Following Response

**DOI:** 10.1371/journal.pone.0260739

**Published:** 2022-09-01

**Authors:** Laís Ferreira, Julia Dalcin Pinto, Déborah Aurélio Temp, Eli Natáli Broman, Piotr H. Skarzynski, Magdalena B. Skarzynska, Denis Altieri De Oliveira Moraes, Milaine Dominici Sanfins, Eliara Pinto Vieira Biaggio

**Affiliations:** 1 Speech Therapy Department, Federal University of Santa Maria, Santa Maria, Brazil; 2 Institute of Physiology and Pathology of Hearing, Warsaw, Poland; 3 Department of Heart Failure and Cardiac Rehabilitation, Warsaw, Poland; 4 Institute of Sensory Organs, Warsaw, Poland; 5 Institute of Sensory Organs, Kajetany, Poland; 6 Center of Hearing and Speech, Kajetany, Poland; 7 Statistics Department, Federal University of Santa Maria, Santa Maria, Brazil; 8 Faculty of Medical Science, State University of Campinas, Campinas, Brazil; 9 Advanced Neuroaudiology and Electrophysiology Center, São Paulo, Brazil; Max-Planck-Institut fur Kognitions- und Neurowissenschaften, GERMANY

## Abstract

During childhood, neuronal modifications occur so that typical childhood communicative development occurs. This work aims to contribute to the understanding of differences in the speech encoding of infants and school-age children by assessing the effects of child development, in different phases of early childhood, on the encoding of speech sounds. There were 98 subjects of both sexes, aged from 1 day to 8 years and 9 months who participated in the study. All subjects underwent a Frequency Following Response (FFR) assessment. A regression and linear correlation showed the effects of age in the FFR components, i.e., significant decrease in the latency and increased amplitude of all FFR waves with age. An increase in the slope measure was also observed. Younger infants require more time and show less robust responses when encoding speech than their older counterparts, which were shown to have more stable and well-organized FFR responses.

## Introduction

The integrity of sensory pathways is fundamental for child development. Processing of auditory input enables the child to discriminate important acoustic cues which culminate in phonological acquisition and improved auditory abilities [1]. Both the peripheral and central auditory pathways play an essential role in the acquisition of oral language.

The processing of speech is a complex process which involves several auditory structures. In this context, the maturation of the Central Auditory Nervous System (CANS) plays an important role in the quality of auditory processing so that, when harmed, can sometimes cause perceptual deficits that may later lead to a communication disorder [2].

CANS maturation is a slow and gradual process, in which full maturity is only reached around adolescence [3–7]. The sensory experiences that the child is exposed to, particularly in the first years of life, modulate the development of the fine neural connections which facilitate speech processing [8]. To adequately diagnose auditory function, a clinician needs to look at all stages of the maturation process.

Assessment of the auditory pathway, particularly in children, must be detailed and sensitive enough to make sure the system is fully able to encode speech. As alternatives for measuring auditory function, evoked potentials, such as the Frequency Following Response (FFR), stand out as one tool that allows the subcortical and cortical structures of the auditory pathway in response to a speech stimulus to be objectively analyzed [9].

The FFR assessment elucidates information related to spectral and temporal processing of verbal sounds in the CANS. FFRs provides a method to assess the encoding of complex sounds such as speech, once it investigates how the speech sounds structure is encoded by the auditory system as a whole [10–12]. Extensive scientific work has pointed to the relevance of the FFR in understanding the mechanisms of speech processing in children’s development [3] and have demonstrated its clinical applicability in different pathologies [13–17]. Such assessments can contribute to the differential diagnosis of several human communication disorders [11, 18].

Early infancy and school age are considered two significant milestones for child development. The first two years of life are a critical period for language acquisition [19] and the first school years mark the beginning of the learning process, particularly acquisition of reading and writing. Although the literature points out that speech encoding and perception can be modulated according to the level of child development [3, 20, 21], few studies have aimed to evaluate such changes during the neonatal and school period. Similarly, although many studies have examined the relationship between CANS maturation and its effect on auditory processing by using auditory evoked potentials, few have made assessments using speech stimuli [2, 3, 18, 20].

Therefore, this study aims to contribute to the understanding of observed differences in the encoding of speech sounds of children throughout different phases of early childhood. In addition, it adds evidence of how CANS maturation modulates speech processing by making FFR assessments at different stages of child development to assess the possible correlation between the FFR variables with age.

## Methods

### Ethical aspects

This research is an observational, analytical, descriptive, and quantitative cross-sectional study approved by the Research Ethics Committee of the Federal University of Santa Maria (number CAAE 81117517.0.0000.5346 and opinion 2.538.043). The norms and regulatory guidelines for research with human beings of Resolution 466/12 of the Brazilian National Health Council were respected. Those responsible for the subjects were informed about the research procedures and those who agreed to participate signed a written consent form. The study was carried out throughout 2019, from January to July.

### Participants and sample procedures

The sample comprised 98 subjects, aged between 1 day to 8.9 years, who were divided into four groups according to their age: Group I (GI), 43 subjects from zero to 30 days (mean age of 15.1 days); Group II (GII), 25 subjects from 31 to 90 days (mean age of 59.1 days); Group III (GIII), 22 children from 5.4 years to 6.1 years (mean age of 6.1 years or 2,220 days); and Group IV (GIV), 8 children from 7.3 to 8.9 years (mean age of 7.8 years or 2,796 days). Infants and children from the Neonatal Hearing Screening (NHS) program and the children’s audiology laboratory of a university hospital participated in the study.

All individuals presented normal newborn hearing screening (NHS) scores, absence of Risk Indicators for Hearing Impairment (RIHI) or neurological deficiency, Apgar scores higher than 8 in the first and fifth minute of life, as well as normal absolute and interpeak latencies of waves I, III, and V of the Auditory Brainstem Response (ABR). In addition, the school-age children (5–8:9 years) needed to have normal auditory thresholds, absence of middle ear alterations, no speech disorders or school complaints, as well as a satisfactory result in a non-instrumental language assessment. Such assessment was performed with the purpose of ruling out any alterations pervading the aspects of syntax, vocabulary, pragmatics and semantics and the outcomes were considered as satisfactory by the absence of phonological errors, articulatory blocks, or reductions in vocabulary.

In order to ensure all participants met the criteria mentioned above, the subjects underwent an ABR recording prior to the FFR assessment to screen for auditory synchrony. In addition, the school-age children underwent a basic auditory evaluation and a language assessment. The ABR was recorded at 80 dBnHL with a click stimulus using the same equipment as used to elicit the FFR [22]. The hearing tests consisted of pure tone audiometry, a speech recognition test, and an acoustic immittance measurement. The information regarding the results from the NHS were extracted from the child’s health passbook. Subjects who met all the criteria proceeded to FFR recording.

### Frequency Following Response

The Smart Ep module from Intelligent Hearing Systems (HIS) was used to record the FFR. In order to perform the exam, the subjects skin was cleaned and the surface electrodes were positioned at Fz, Fpz, M1, and M2. Impedance was maintained at <3k ohms, with a difference of <2 ohms between positions.

The FFR was recorded with speech stimuli, represented by the syllable /da/, of 40 ms duration, which was presented to the subjects through insert headphones. The stimuli were presented at a rate of 10.9/s with alternating polarity. The analysis window was 80 ms and a high-pass filter of 100 Hz and a low-pass of 2000 Hz were used. Runs with more than 5% artifacts were excluded. The protocol adopted to register the FFR responses was chosen due to current literature on the topic [23].

**Fig 1:**
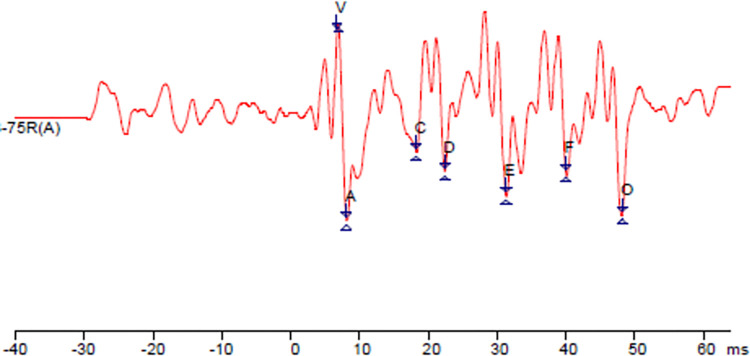
Representative illustration of the neural response evoked by the Frequency-Following Response of a six-year-old child. ms = milliseconds. Source: Author’s data. FFR performed using Smart EP. The labels in the waveform mark the FFR waves (V, A, C, D, E, F and O).

The FFR assessment used two sweeps of 3000 stimuli. The sweeps were summed to generate a resultant trace, on which the waves V, A, C, D, E, F, and O were marked, as illustrated in Fig 1. The markings were done according to an international reference [3]. In this way, the absolute latencies, amplitude, and slope were extracted from each trace. It’s relevant to add that latency reflects the duration it takes to generate the neural responses, amplitude is the measure portraying the synchrony of the responses and, the slope corresponds to the relation between the time and the magnitude of the neural response of the VA complex [23].

To capture the FFR responses of infants, they were recorded in natural sleep in a quiet room, comfortably accommodated in the lap of their carer while the examinations were done. With the school-age children, assessments were done while the child sat, awake and calm, in a comfortable reclining armchair.

### Statistical analysis

The variables analyzed in this study were: absolute latencies and amplitudes of waves V, A, C, D, E, F, and O and the slope measure. The variables were extracted from the registers and the results tabulated in a spreadsheet. A multivariate linear regression with dummy variables to model the age variable into four groups were performed to analyze the dependence between the absolute latencies and amplitudes of waves as well as the slope measure in time. The estimated coefficients were evaluated by a 5% significance level. P-values lower than 0.05 were significant.

The variable Age is presented in days and then categorized in groups for further analyzes by the multivariate regression model. To correctly use the regression model, we create three indicators variables (dummy) as defined in the Table 1 below. So, a GI individual must have 0 values for D1, D2 and D3 simultaneously, and a GII individual is the case where only D1 is set to 1, while keeping D2 and D3 fixed as 0 and so on.

**Table 1:** Definition of dummy variables for age group from age as categorical variable.

Group\Dummy	D1	D2	D3
GI	0	0	0
GII	1	0	0
GIII	0	1	0
GIV	0	0	1

In this sense, all state comparisons are regarded to the base category, which is the GI. The base effect of the group GI is accounted in the Intercept coefficient, meaning that the GII coefficient is added do the Intercept coefficient to measure the overall response effect in the state of group GII, as well as the GIII and GIV coefficients, which are added to the Intercept of the model, or the GI effect. The estimated model then can be written in the form:

$${\hat{Y}}_{i}={\hat{\beta }}_{0}+{\hat{\beta }}_{1}D1+{\hat{\beta }}_{2}D2+{\hat{\beta }}_{3}D3$$

If an individual belongs to GI, then D1 = D2 = D3 = 0 and *Y* = *β*_0_, which means the base category effect. If the individual belongs to GII, then (${\hat{Y}}_{i}=\beta$), which adds *β*_1_ for the base effect. In the same way, for GIII individuals the predicted value for the dependent variable is (${\hat{Y}}_{i}=\beta$) and for a GIV member the response is (${\hat{Y}}_{i}=\beta$). This method of comparison gives a fair coefficient for the current group when compared to the other groups, as the reference point is the same and despite the proximity between the groups.

## Results

The mean values of latency, amplitude, and slope are described in Table 2 for group GI, GII, GIII and GIV.

**Table 2:** Descriptive values of the Frequency-Following Response variables for groups I, II, III and IV.

Group	GI		GII		GIII		GIV	
Variable	Mean	SD	Mean	SD	Mean	SD	Mean	SD
Age	15.3	7.6	49.1	20.7	2176.4	131.0	2902.5	240.5
Lat_V	8.0	0.6	7.5	0.4	6.8	0.3	6.8	0.3
Lat_A	9.4	0.6	8.5	0.5	7.9	0.3	7.8	0.4
Lat_C	18.7	1.2	17.9	1.1	17.4	1.3	17.5	1.0
Lat_D	24.4	1.0	23.9	0.6	23.1	0.9	22.9	0.8
Lat_E	33.4	1.1	32.1	1.0	32.1	1.1	32.1	0.9
Lat_F	41.2	1.2	40.4	0.6	40.0	0.7	40.0	1.0
Lat_O	49.4	0.6	48.8	0.5	48.0	0.6	48.0	0.4
Slope	0.3	0.1	0.5	0.1	0.5	0.2	0.6	0.3
Amp_V	0.2	0.1	0.2	0.1	0.2	0.1	0.3	0.1
Amp_A	-0.2	0.1	-0.2	0.1	-0.3	0.1	-0.3	0.1
Amp_C	-0.1	0.2	-0.1	0.1	-0.2	0.1	-0.2	0.1
Amp_D	-0.1	0.1	-0.1	0.1	-0.3	0.1	-0.2	0.1
Amp_E	-0.2	0.1	-0.2	0.1	-0.2	0.1	-0.3	0.1
Amp_F	-0.2	0.1	-0.2	0.1	-0.3	0.2	-0.3	0.2
Amp_O	-0.2	0.1	-0.2	0.1	-0.3	0.2	-0.3	0.1

Table 3 shows the regression analysis coefficients for age group effects in the latency variables. The estimated coefficients were significant for all the FFR component models. By this result, we may infer that the neural conduction time represented by the FFR waves suffer from the age impact when the GII, GIII and GIV are compared to the base state, which is the GI. The highest coefficient of determination was observed in the Lat A component (r^2^ = 0.627), meaning that 62.7% of the component variability was explained by age in the form of categorical variable.

**Table 3:** Regression analysis of the latency in Frequency-Following Response components in relation to age group I, II, III and IV.

FFR	Coefficients	Estimate	P-value
Lat V	Intercept	7.98	0.000
	GII	-0.49	0.000
Adj.R^2^ = 0.553	GIII	-1.20	0.000
	GIV	-1.15	0.000
Lat A	Intercept	9.37	0.000
	GII	-0.84	0.000
Adj.R^2^ = 0.627	GIII	-1.51	0.000
	GIV	-1.56	0.000
Lat C	Intercept	18.74	0.000
	GII	-0.86	0.004
Adj.R^2^ = 0.180	GIII	-1.32	0.000
	GIV	-1.27	0.005
Lat D	Intercept	24.42	0.000
	GII	-0.50	0.023
Adj.R^2^ = 0.289	GIII	-1.28	0.000
	GIV	-1.51	0.000
Lat E	Intercept	33.39	0.000
	GII	-1.29	0.000
Adj.R^2^ = 0.254	GIII	-1.31	0.000
	GIV	-1.30	0.002
Lat F	Intercept	41.18	0.000
	GII	-0.79	0.001
Adj.R^2^ = 0.227	GIII	-1.22	0.000
	GIV	-1.20	0.001
Lat O	Intercept	49.42	0.000
	GII	-0.67	0.000
Adj.R^2^ = 0.508	GIII	-1.44	0.000
	GIV	-1.45	0.000

Lat = Latency; GI = newborns; GII = infants; GIII = preschool children; GIV = school aged children; Adj. R^2^ = model adjusted coefficient of determination.

For the analysis of the magnitude of the neural response, the absolute amplitude values were obtained. Table 4 shows that although almost all regression coefficients of FFR amplitudes are statistically significant, the adjusted r^2^ measures are lower in general in these components than latency. In some cases, as in Amp C, the change in GI to GII does not demonstrate statistical difference (p = 0.915) but the change from GI to GIII does (p = 0.003). In this case, also observe that with the low value of the adjusted r^2^ (8.1%), there was no statistical impact of GIV coefficient over the Intercept of the model (p = 0.167).

**Table 4:** Regression analysis of the Frequency-Following Response amplitude components in relation to age group.

FFR	Coefficients	Estimate	P-value
Amp V	Intercept	0.16	0.000
	GII	0.08	0.000
Adj. R^2^ = 0.189	GIII	0.08	0.000
	GIV	0.11	0.001
Amp A	Intercept	-0.17	0.000
	GII	-0.07	0.002
Adj. R^2^ = 0.261	GIII	-0.13	0.000
	GIV	-0.12	0.001
Amp C	Intercept	-0.09	0.000
	GII	0.00	0.915
Adj. R^2^ = 0.081	GIII	-0.10	0.003
	GIV	-0.07	0.167
Amp D	Intercept	-0.12	0.000
	GII	-0.03	0.288
Adj. R^2^ = 0.199	GIII	-0.13	0.000
	GIV	-0.07	0.080
Amp E	Intercept	-0.18	0.000
	GII	-0.03	0.303
Adj. R^2^ = 0.033	GIII	-0.06	0.029
	GIV	-0.07	0.100
Amp F	Intercept	-0.15	0.000
	GII	-0.06	0.062
Adj. R^2^ = 0.135	GIII	-0.11	0.001
	GIV	-0.15	0.002
Amp O	Intercept	-0.15	0.000
	GII	-0.06	0.067
Adj. R^2^ = 0.275	GIII	-0.18	0.000
	GIV	-0.17	0.001

Amp = Amplitude; GI = newborns; GII = infants; GIII = preschool children; GIV = school aged children; Adj. R^2^ = adjusting degree.

**Fig 2:**
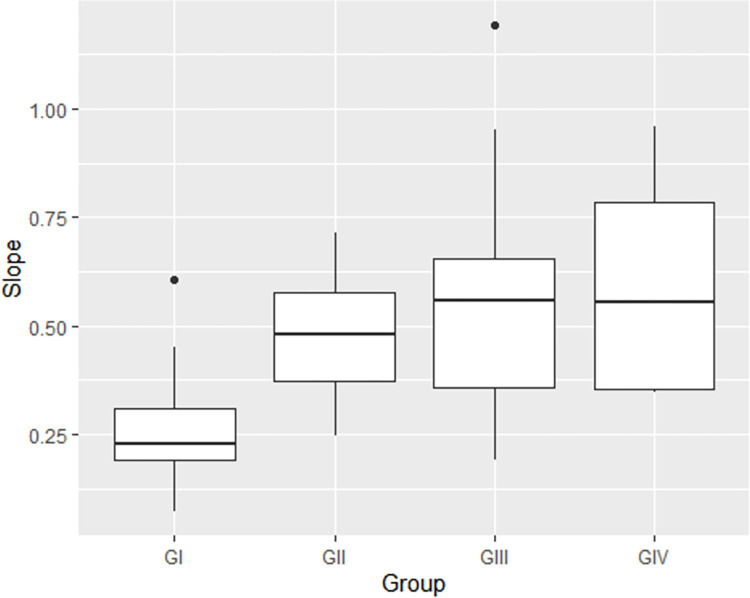
Boxplot of the slope distribution in function of age groups. GI = newborns; GII = infants; GIII = preschool children; GIV = school aged children. P-value = 0.000; Adj. R^2^ = 0.401.

Fig 2 depicts the slope distribution characteristics in the four age groups, i.e., the synchronism of the FFR response generators in time. The results in Table 5 obtained by the multiple regression adjusted coefficient of determination (r^2^ = 0.401) show that of the total variation occurring in the slope, 40% may be explained by the age group variability, the remaining and not accounted variability sources are possibly due to individual characteristics or random variables that could not have been particularly assessed.

**Table 5:** Regression analysis of the Frequency-Following Response slope components in relation to age group.

FFR	Coefficients	Estimate	P-value
Slope	Intercept	0.25	0.000
	GII	0.22	0.000
Adj. R^2^ = 0,401	GIII	0.29	0.000
	GIV	0.35	0.000

## Discussion

This study contributes to evidence that FFR responses are affected by the level of child development, particularly correlated with the variable of age [3, 20]. It provides data on functional modifications to the CANS which arise from maturation of auditory pathways, showing how these are reflected in neuroelectrophysiological measures. Thus, it was observed that during infancy and across childhood, subjects underwent physiological changes in the auditory pathway, which may explain the expected better outcomes evidenced in the FFR assessment as the child develops, i.e., decreasing neural conduction time and increasing the magnitude of all FFR waves.

Long before birth, infants are able to perceive auditory stimuli and many events act on the prior immature but highly plastic brain promoting its development [1, 4, 5]. Measures derived from auditory evoked potentials, such as the ones assessed by the FFR, are useful tools as they provide a closer look into the neural processes involved in speech processing [5], from perceiving the sensory signal to encoding the acoustic cues, and later performing higher levels associations between these different types of information.

The regression analysis performed in this study showed correlations of all FFR variables, i.e., latency, amplitude and slope, with age (Tables 2–4 and Fig 2). These results clearly demonstrate that significant modifications occur in FFR responses in the first months of life, showcasing the maturation of the CANS and of how the infants perceive speech sounds, continuing to progressively develop until adolescence [4, 24]. In line with these data, researchers who have studied FFRs across the lifespan have observed that neural changes in the school period are less intensive than during infancy and that, by early adulthood, neural development of the auditory structures tends to stabilize alongside the neural responses assessed by this procedure [3].

Like the results of this research, previous authors have also found evidence that FFRs are affected by age [3, 18, 20, 25], majorly affecting the latency values. The findings from our study as well as the studies mentioned above point to the idea that younger infants require more time to involuntarily process the auditory information as they are initially learning to discriminate phonological cues and associate it with the respective semantic correlate, whereas older children, such as the ones already exposed to school and further stimuli-rich environments, show a much more stable and well-organized neural circuit to account for encoding and processing speech sounds [26].

Further research on the topic has made much clearer the relationship between the innate factors, i.e., the biological substrates, and the external factors, such as the language experiences involved in speech processing. Jeng *et al* have shown that neonates are able to detect differences in voice pitch similarly regardless of the language of the stimuli used to evoke their responses, which shows the maturation of the brainstem structures at ages as early as 1 to 3 days and that, by adulthood, this processing system likely differ as an adaptation response to the environmental stimuli [27].

Our analysis demonstrated decreased neural conduction time, increased magnitude of neural responses, and increased slope with age. In general, children have lower latency and higher amplitudes compared to infants. A reduction in latency with age has also been observed in other studies performed with the FFR [3, 18, 20, 25], but few were the papers to examine the absolute amplitude values of the FFR waves. Our study is therefore unique in pointing to the effects of maturation on neural networks associated with the encoding of speech.

Considering that the slope assesses the existing relation between the latency and amplitude responses, the observed results for the slope values, i.e., increase with age (Fig 1), suggests that children, compared to infants, exhibit an improved time to magnitude ratio of the neural response of the VA complex.

Other research involving children has demonstrated similar results to those observed here. In addition to observing a decrease in latency with age, authors report better FFR responses, both in the time and frequency domains, during childhood, i.e., improved outcomes over the course of infancy and early childhood [3, 18, 20, 25]. This is probably the result of age-related advancement in neural development, so that the brain areas responsible have a better level of functional development [3, 20].

The subcortical structures undergo rapid structural growth, with an increase in the myelin density of axons in these regions [4, 28]. This growth progresses until about 12 years of age, at which time the density of axons approaches the adult pattern [4, 28]. Several authors have attributed improved FFR results with age to myelination and synaptic specialization [3, 20], suggesting that a faster and more synchronized neural conduction is part of its lifelong development [4, 24, 28, 29].

Associated with these physiological processes, environmental experiences also play an important role in child development [30, 31]. Exposure to environmental stimuli promotes rapid maturational development of the child’s central nervous system [8, 32]. Auditory experience improves synaptic connections, neural specialization, and more effective synapses [4, 33]. Also, it’s important to note that the listener’s linguistic experience has been shown as one major factor influencing the whole mechanism underlying the processing of voice pitch [27].

Although we could not determine the magnitude of the environmental stimuli effect on the improvement of the FFR responses throughout child development, this variable must be emphasized as it is well known that exposure to environments rich in sensory stimuli is associated with several neural changes, such as increased cortical circumference and thickness, dendritic ramifications, and synaptic density [24], and therefore may have contributed to the improved FFR responses with the advancing of age.

The idea of age-related changes in early and late auditory responses, such as those seen in brainstem and cortical stimulation, is well established in the literature, however, the same cannot be said for subcortical assessments. Considering those neural areas responsible to evoke the FFR response and given that recent research has found that FFR assessment may be a useful tool in predicting later impaired language development [34], understanding auditory maturation and its effects on the FFR is of great interest, and requires close definition of the parameters used in clinical assessments.

Research to date has demonstrated the feasibility of successfully recording FFR responses in children; however, as most studies show, the response patterns change significantly according to the specific population tested. Here we have shown the differences observed in a large sample of children of different ages, showing how FFR responses–latency, amplitude, and slope–change throughout a child’s development. These measures provide useful data regarding recruitment and the neural substrates responsible for auditory and speech encoding. Further analysis, such as a frequency analysis, might be of benefit in providing more detailed evidence.

## Conclusion

Neural encoding of speech undergoes changes during child development. Increasing age and the associated central auditory nervous system development contribute to an improvement in the performance of the neural network responsible for the encoding and processing of sounds. The physiological changes that occur across childhood may be observed in the FFR assessment, procedure which shows great promise to be used as a biological marker of auditory pathway maturation.

## Supporting information

S1 Data (XLSX)
